# From Nonfunctioning Adrenocortical Cancer to Biochemically Silent Paraganglioma Associated with *SDHB* Mutation: An Uncommon Presentation of a Patient with a Retroperitoneal Mass

**DOI:** 10.1155/2024/6664694

**Published:** 2024-08-02

**Authors:** Izabella Freitas, Anna Albuquerque, Luiz de Marco, Eduardo, José Renan Melo, Juliana Drummond, Beatriz Rocha

**Affiliations:** ^1^ Department of Endocrinology Federal University of Minas Gerais (UFMG), Belo Horizonte, Minas Gerais, Brazil; ^2^ Department of Molecular Medicine UFMG, Belo Horizonte, Minas Gerais, Brazil; ^3^ Department of Pathology UFMG, Belo Horizonte, Minas Gerais, Brazil; ^4^ Department of Surgery UFMG, Belo Horizonte, Minas Gerais, Brazil

## Abstract

The combination of clinical characteristics and diagnostic exams including imaging, laboratory, and molecular tests help in the differential diagnosis of retroperitoneal lesions. We report a 41-year-old male with a metastatic retroperitoneal lesion with atypical characteristics, displaying pathological findings consistent with both nonsecretory pheochromocytomas/paragangliomas and adrenal cortex carcinoma. The patient was examined for abdominal pain, weight loss, and hypertension. Abdominal computed tomography showed a 21 × 8 × 10-cm right retroperitoneal mass. He was initially diagnosed as pheochromocytoma/paraganglioma (PHEO/PGL). However, the diagnosis was later changed to adrenocortical carcinoma based on histopathological features of the metastatic lesions and the findings of normal urinary levels of catecholamines/metanephrines. Systemic chemotherapy and abdominal radiotherapy were performed, in addition to multiple surgical resections, with no satisfactory response. The indolent course of the disease and minimal impact on the patient's performance status led to a genetic evaluation which resulted in the identification of a germline mutation in the succinate dehydrogenase complex subunit B (*SDHB*). An immunohistology review of previous slides was consistent with the hypothesis of a neuroendocrine tumor. Forty percent of the patients with PHEO/PGL have an underlying germline mutation. *SDHB* mutation is frequently associated with metastatic disease and dominant secretion of noradrenaline and/or dopamine. In addition to the metastatic disease, few cases with the mutations can be a biochemically silent PHEO/PGL. We concluded that the patient presented a metastatic abdominal paraganglioma associated with an *SDHB* mutation and we reinforced the need to perform genetic screening for all adrenal/extra-adrenal lesions characteristic of PHEO/PGL.

## 1. Introduction

Pheochromocytomas and paragangliomas (PHEO/PGL) are rare neuroendocrine tumors. From 80% to 85% of cases are tumors arising from adrenal medulla and 15% to 20% of cases are tumors of paraganglioma arising from extraganglioma. Typically, PHEO/PGL secrete catecholamines or their metabolites and the patients mainly present characterized symptoms such as hypertension, sweating, headache, and palpitations. A “biochemically silent” PHEO/PGL is rare and in these cases, the patient's clinical presentation may be secondary solely to the mass effect caused by the tumor [1].

Most PHEO/PGL are sporadic, but approximately 40% of the cases are associated with germline pathogenic variants. The main PHEO/PGL susceptibility genes are *SDHB*, *SDHD*, *VHL*, *NF-1*, and *RET* [1]. The succinate dehydrogenase (*SDH*) enzyme complex is a hetero-oligomer comprising four structural subunits: *SDHA*, *SDHB*, *SDHC*, and *SDHD*. In the Krebs cycle, *SD*H catalyzes the oxidative dehydrogenation of succinate to fumarate. Loss-of-function mutations in the *SDHx* genes can lead to enzyme inactivation and cellular accumulation of succinate, which is considered an oncometabolite [2, 3].

The diagnosis of PHEO/PGL is established based upon the laboratory finding of excess catecholamines or their metabolites followed by identification of the tumor in imaging exams. Treatment of patients with localized disease consists of surgical resection of the tumor and management of adrenergic symptoms [1]. Only 15%–17% of PPGL present malignant behavior, defined by the presence of metastases [1]. Factors leading to a worse prognosis are *SDHB/SDHD* gene mutations, primitive tumor >5 cm, extra-adrenal location, age at diagnosis <50 years, dopaminergic phenotype (increased plasma methoxytyramine levels), and high Ki-67 index [1]. Treatment of patients with metastatic disease includes alpha-blockers in the case of functioning PHEO/PGL, surgical resection, and local therapies (radiotherapy and radiofrequency ablation) if feasible, systemic treatment to control tumor growth, and antiresorptive therapy for bone metastases [1]. Molecular targeted therapy is increasingly being studied in patients with metastatic PHEO/PGL and may play an important role in the future treatment of these tumors [4].

Among retroperitoneal tumors, aside from PHEO/PGL, adrenal cortex carcinoma (ACC) needs to be considered in the differential diagnosis. These tumors are also rare and extremely aggressive. Most cases occur sporadically but may also be present in the context of genetic syndromes, mainly Li–Fraumeni syndrome, Beckwith–Wiedeman syndrome, Lynch syndrome, and Multiple Endocrine Neoplasia Type 1. Clinical manifestations frequently result from the hypersecretion of cortisol and/or androgens. Apparently, nonfunctioning ACCs are uncommon [5]. Histopathological diagnosis through the use of the Weiss criteria is decisive for the final diagnosis and may help in evaluating prognosis. Immunohistochemistry profile is essential to characterize the adrenocortical origin of the tumor. Positive immunoreactivity for Melan-A, steroidogenic factor (SF-1), and alpha-inhibin confirms the adrenocortical origin of the lesion, while positive immunoreactivity for Chromogranin A rules out this origin [6]. Standard treatment consists of surgical resection, chemotherapy, and radiotherapy. In most cases, patients have a very poor prognosis [5].

We present a case of a 41-year-old man, with a metastatic retroperitoneal lesion with atypical characteristics. After extensive review, we concluded that the patient presented a biochemically silent metastatic abdominal paraganglioma associated with an *SDHB* germline mutation.

## 2. Case Presentation

A 41-year-old male, rural worker, was examined in 2014 for abdominal pain, weight loss, and report of increased blood pressure levels, without experiencing paroxysms. He denied taking any medications and had no previous history of other comorbidities. The patient had no family history of systemic arterial hypertension with associated secondary causes. His mother died at the age of 65 due to an abdominal cancer of unknown primary site.

On physical examination, he was normotensive with a normal heart rate. A palpable mass was identified in the upper abdomen. Abdominal computed tomography (CT) showed a 21 × 8 × 10-cm right retroperitoneal mass attached to the inferior vena cava, upper pole of the right kidney, and right lobe of the liver. Complete blood count and serum levels of electrolytes, glucose, kidney, and liver functions were normal. Preoperative plasma and urinary metanephrines and catecholamines levels were not measured at that time because the patient was managed by the general surgery team and the hypothesis of PHEO/PGL had not been considered.

He underwent tumor excision, including right adrenalectomy in March 2014. The anatomopathological results were consistent with epithelioid cell neoplasia (possibly alveolar soft part sarcoma, renal cell carcinoma, paraganglioma, or adrenocortical neoplasia). Immunohistochemistry suggested neuroendocrine neoplasia: cytokeratin+, chromogranin A+, S100+, vimentin+, and Ki67 of 3%. Owing to the tumor location and morphology, the possibility of PHEO/PGL was raised. For that reason, urinary hormone workup was performed in the postoperative period, showing normal levels of results for catecholamines and metanephrines.

Six months later, he began experiencing right lower back pain, vomiting, and tachycardia. Radiological recurrence of the lesion was identified revealing a retroperitoneal mass of 8 cm. The urinary workup was, again, negative for catecholamine secretion. A diagnosis of recurrent PHEO/PGL was suggested, with the possibility of intermittent catecholamine release. A new tumor resection was performed in February 2016. At that time, anatomopathological results were suggestive of adrenal cortical carcinoma. The use of few immunohistochemical markers is noteworthy, as the tumor was negative for cytokeratin, synaptophysin, and CD34, with Ki67 of 5%.

In May 2016, he underwent metaiodobenzylguanidine scintigraphy (I-123-MIBG), which revealed the absence of anomalous chromaffin tissue throughout the body. Hormonal hypersecretion was ruled out: testosterone, dehydroepiandrosterone sulfate, cortisol, plasma renin activity, aldosterone, and urinary metanephrines/catecholamines levels were within normal range. However, chromogranin A was 1.8 times above the upper normal limit (reference value 100 ng/mL). A new CT scan of the chest revealed pulmonary nodules with metastatic characteristics. Immunohistochemistry examination of pulmonary metastasectomy was positive for calretinin, favoring the diagnosis of ACC over the hypothesis of PHEO/PGL, although calretinin positivity is not specific for ACC.

**Figure 1:**
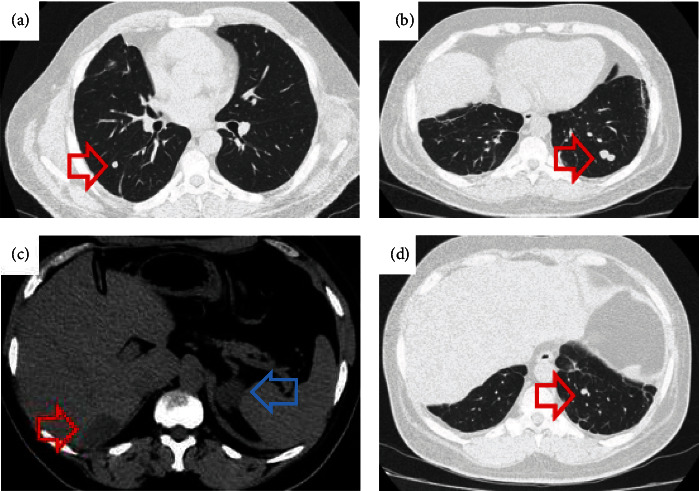
(a) Pulmonary metastasis on CT, June 2020. (b) New foci of pulmonary metastasis on CT, April 2021. (c) Liver metastasis on CT, December 2021, approximate size of 3.9 × 2.2 cm (red arrow). Left adrenal nodule (blue arrow). (d) New foci of pulmonary metastasis on CT, December 2021.

Chemotherapy (cisplatin, etoposide, and doxorubicin) was administered from September 2016 until February 2017. Mitotane was prescribed between 2016 and 2019. During follow-up, the disease progressed (Figure 1) with new surgical approaches for the resection of abdominal and pulmonary nodules. An [18F]-fluorodeoxyglucose positron emission tomography and computed tomography scan (^18^F-FDG PET/CT) performed in August 2017 highlighted pulmonary, hepatic, and retroperitoneal lesions. Faced with the refractoriness to the instituted treatment, abdominal radiotherapy was performed empirically from January 2018 until March 2019.

**Figure 2:**
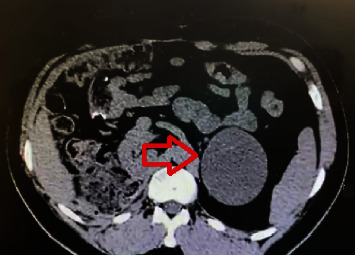
On the red arrow, a focal lesion in the left adrenal gland with well-defined limits and 8 HU density in December 2021. Approximate size of 7.9 × 6.7 cm.

In 2021, a 3-cm nodule was identified in the left adrenal gland. After 8 months, the tumor had grown to 5 cm, and maintained a regular appearance, without invasive features (Figure 2). In January 2022, he underwent left adrenalectomy. The neoplasm's histological pattern had altered, with some areas showing mucin-like material deposits, which did not meet the Weiss criteria [6] for adrenal carcinoma. The patient later underwent two sessions of percutaneous ethanol embolization for the treatment of liver metastases in 2022.

In November 2022, DNA from peripheral blood and paraffin-embedded from three different sites and dates (left adrenal, right adrenal, and psoas) were extracted. Genomic DNA from peripheral blood was subjected to whole-exome capturing and sequencing with average coverage of 100x using the Illumina DNA Prep with the Illumina NovaSeq 6000 sequencing platform (San Diego, CA 92122). A heterozygous pathogenic variant (PV) of the *SDHB* gene in exon 6 (chr1:17350519) was identified and validated using Sanger sequencing (*Supplementary Figure 1*). It is a single nucleotide deletion (rs1060503757/c.591del/p.Ser198Alafs ^*∗*^), which causes a frameshift and generates an early stop codon in the main transcript (ENST00000375499), resulting in a truncated protein that differs from the wild-type *SDHB* protein by 21 amino acids and is shortened by 62 residues. We demonstrate in silico models of the wild-type SDHB protein and mutated SDHB protein (*Supplementary Figure 2*). SDHB protein sequence was obtained at UniProt [7] and used to build protein model of both the wild-type and predicted pathogenic mutated protein. To evaluate the loss of heterozygosity, DNA from all three tumor tissues was subjected to Sanger sequencing, which confirmed the PV in homozigosity in all tumor samples. Subsequently, genetic screening of family members demonstrated the presence of this variant, in heterozygosis, in the eldest son. Variant pathogenicity was assessed using ACMG classification [8] and the pathogenicity scores performed using Mutation Taster [9], PolyPhen [10], SNPs&Go [11], SIFT [12], PhdSNP [13], PANTHER [14], SNAP2 [15], PROVEAN [16], and Human Splicing Finder Pro Version 4.3.2 software [17].

**Figure 3:**
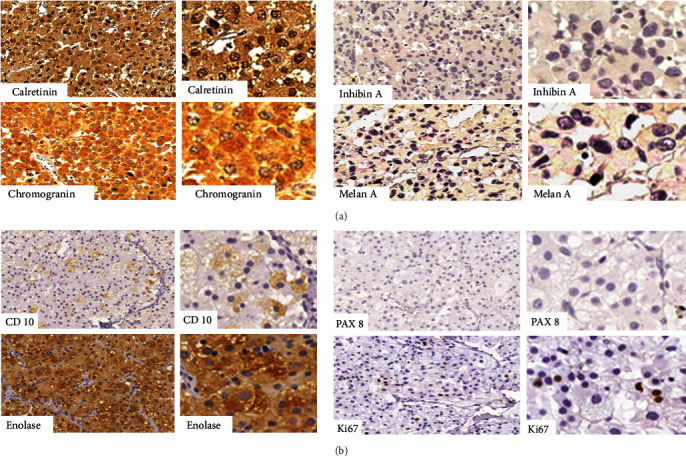
(a) Retroperitoneal nodule resection (2016): Immunohistochemistry shows strong positivity for calretinin and chromogranin, and negativity for inhibin-A and melan-A. (b) Pulmonary metastasectomy (2021): Immunohistochemistry shows strong positivity for enolase, negativity for CD10 and PAX 8, and weak positivity for Ki67 (3%). These results support the diagnosis of a neuroendocrine tumor as opposed to a cortical or renal tumor.

An experienced pathologist from an academic center reviewed the histopathological slides in 2022. From a histological perspective, the tumor changed characteristics over the years but maintained the findings of little cellular atypia, rare mitotic figures, and absence of angiolymphatic invasion (*Supplementary Figure 3*). The hypotheses of neuroendocrine tumors, adrenal cortex tumors, and even oncocytic renal carcinoma based on histology were suggested. However, in immunohistology, features of neuroendocrine tumors predominated, and the hypothesis of PHEO/PGL was primarily considered (Figures 3(a) and 3(b)). It is worth mentioning that in this review carried out in 2022, the pathologist added the analysis of other immunohistochemical markers in the sample from the 2016 surgery specimen that had not been carried out at the time of diagnosis (*Supplementary Table 1*).

**Figure 4:**
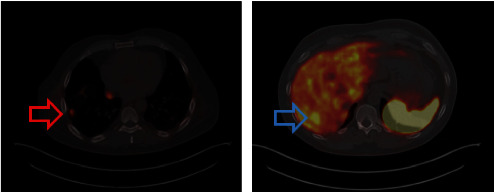
Overexpression of the radiopharmaceutical (NOTANOC-18F) in a pulmonary (red arrow) and hepatic nodule (blue arrow).

**Figure 5:**
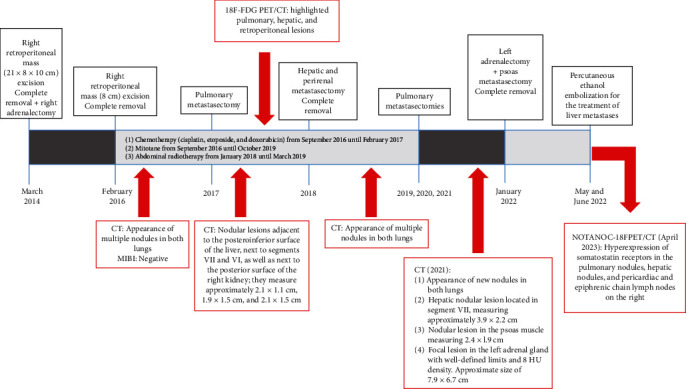
Timeline of the main treatments instituted between 2014 and 2023. The red arrows show the major image findings.

The patient underwent [18F]-NOTANOC positron emission tomography and computed tomography scan (NOTANOC-^18 F^ PET/CT) in April 2023, which showed hyperexpression of somatostatin receptors in the pulmonary nodules, hepatic nodules, and pericardiac and epiphrenic chain lymph nodes on the right (Figure 4). Currently, the patient is taking prednisone and fludrocortisone, maintains clinical stability, and actively works in rural activities. We present a timeline of the main treatments instituted between 2014 and 2023 in Figure 5.

## 3. Discussion

In the clinical case here presented, despite the controversy between initial histopathological reports, the aggressive nature of the retroperitoneal mass was evident. Nevertheless, the indolent course of the disease and the minimal impact on the patient's performance status led us to reconsider the diagnosis of a metastatic adrenal carcinoma in view of the usual poor prognosis of affected patients: Would the tumor be a less aggressive variant of an adrenal carcinoma? Could it tumor be a neuroendocrine tumor originating from chromaffin cells? Consequently, molecular evaluation of blood and tumor samples was performed, revealing an *SDHB* mutation.

Forty percent of the patients with PHEO/PGL carry a germline mutation. All patients with PGL/PHEO should undergo genetic screening. Once a pathogenic variant is identified, germline genetic testing should be offered to all first-degree family members of affected individuals. Clinical penetrance is variable; however, a positive screening test in asymptomatic patients allows longitudinal follow-up, aiming at a possible early diagnosis of the neoplasm [1].

Certain features of *SDHB*-related PHEO/PGLs may be highlighted. These tumors are characterized by lower tumoral catecholamine content and predominantly secrete noradrenaline and dopamine. The lifetime penetrance of *SDHB*-related PHEO/PGLs is clearly superior to the penetrance of *SDHA*- and *SDH*C-related PHEO/PGLs. Metastatic disease is described in 38%–85% of affected patients, depending on the studied population [18, 19, 20]. Interestingly, mutations that result in truncated proteins, as illustrated by the present case, are also associated with an increased risk of malignancy compared with missense variants [21]. Another important feature is the low sensitivity of radioiodine-labeled MIBG scintigraphy in identifying metastases. ^18^F-FDG PET/CT has better accuracy; however, the most accurate functional imaging modality in these cases is PET/CT with somatostatin analogs labeled with gallium^68^.

When we collectively consider PHEO/PGLs, the minority of those arising below the neck are nonfunctional and can be called biochemically silent. It should be noted, however, that parasympathetic (anterior thoracic and head and neck) PPGLs are predominantly nonfunctional. Most cases of biochemically silent PHEO/PGLs reflect small tumors in which metabolite production is insufficient, very large tumors with increased intratumoral metabolism of metanephrines, metastatic disease with some degree of cellular dedifferentiation or exclusive production of dopamine, in which case the measurement of its metabolite, 3-methoxytyramine, is not widely available. This dedifferentiation process may be associated with the absence of tyrosine hydroxylase that limits the initial rate of catecholamine synthesis [22]. In these cases, the diagnosis is usually made based on the mass effect due to the large volume of the tumors. Clinically, dopamine-producing tumors may be associated with atypical symptoms such as hypotension, tachycardia, and polyuria [23]. In cases of silent phenotypes, circulating chromogranin A may be a useful marker [24]. For instance, in the clinical case here presented, the patient had some episodes of postural hypotension during hospitalizations, but confounding factors such as anemia and hypovolemia were also present. Regarding the findings of the last adrenalectomy, a mucinous pattern has been previously described in the literature as suggestive of carcinomatous differentiation [6].

The etiological diagnosis of a retroperitoneal tumor can be challenging. In addition to mesenchymal tumors, adrenal, renal, pancreatic, gastrointestinal, germ cell, and lymphoproliferative lesions need to be considered in the differential diagnosis. The distinction between ACC versus PGL/PHEO may be difficult. In doubtful cases, immunohistochemistry for SF1 is the most sensitive and specific marker available to establish if the tumor is of adrenocortical origin, with a sensitivity of 98% and a specificity of 100% [25]. However, the literature also suggests that the diagnosis of a tumor of adrenocortical origin should be based on a combination of markers, which should include inhibin, melan-A, and calretinin [26]. Depending on the differential diagnosis, other immunohistochemistry markers should be used and alternative diagnoses may be considered. For example, PAX8, which is a useful marker for renal neoplasms, is only rarely positive in ACC.

Zhang et al. [27] reported positive immunoreactivity for calretinin, melan-A, and inhibin in 25%, 5%, and 16% of 20 evaluated PPGLs, respectively. A previous study underscored the complexity that may arise in the distinction between adrenal cortical lesions and pheochromocytoma, based on overlapping morphologies as well as some degree of immunophenotypic overlapping, including focal staining with markers of purported lineage specificity [28]. The authors concluded that using an immunohistochemical antibody panel consisting of chromogranin A plus the nuclear antibody SF-1 and either calretinin or inhibin, while requiring a high-staining intensity threshold, could help to eliminate interpretative issues of artifactual or background reactivity and would improve diagnostic sensitivity/specificity in the differentiation of adrenal cortical lesions from PGL/PHEO. In our case, the combination of immunohistochemistry markers suggested a neuroendocrine origin of the tumor. There was weak reactivity for some adrenal cortex tumor markers, which has been well described in the literature in cases of PGL/PHEO [27], but at some point, brought uncertainty to the diagnosis. Nevertheless, an association between the two tumor etiologies was definitely ruled out.

## 4. Conclusion

We concluded that the patient presented a metastatic abdominal paraganglioma associated with an *SDHB* mutation. The retroperitoneal location of the first excision suggested an extra-adrenal location. Regarding the functionality of the tumor, we believe that it displays either a silent or a dopamine phenotype, for which the measurement of 3-methoxytyramine was not available at our hospital. Further hypotheses for the atypical presentation include tumor dedifferentiation and/or reduction in hormone production after prolonged treatment over these 9 years. This study was conducted in a public hospital with limited financial resources. In addition to the atypical presentation of the case, the unavailability of certain immunohistochemical markers and the limited access to modern nuclear medicine diagnostic tests may have contributed to a delay in the correct diagnosis.

Our treatment proposal is now to progress to radioisotope therapy using Lutetium-177-labeled peptide receptor radionuclides. While awaiting the approval of the therapy by the healthcare system, the patient was started on octreotide 20 mg every 28 days. The asymptomatic family member carrying the *SDHB* mutation will be followed with clinical evaluation, laboratory tests including basic biochemistry and measurement of plasma metanephrines, and radiological/functional imaging studies including MRI from the base of the skull to the pelvis and PET-CT [29].

## Data Availability

All data generated or analyzed during this study are included in this published article and its supplementary materials files.

## Supplementary Materials

Supplementary 1 Figure 1: sanger sequencing chromatogram from blood sample.

Supplementary 2 Figure 2: in silico model of wild-type and mutated SDHB protein.

Supplementary 3 Figure 3: histological appearance of right retroperitoneal tumor (2014) and right perirenal lesion (2018).

Supplementary 4 Table 1: panels of immunohistochemical markers.
