# Supplementary material for: From Nonfunctioning Adrenocortical Cancer to Biochemically Silent Paraganglioma Associated with SDHB Mutation: An Uncommon Presentation of a Patient with a Retroperitoneal Mass
Source: Case Rep Endocrinol. 2024 Aug 2;2024:6664694. doi: 10.1155/2024/6664694 (PMC11315972; doi:10.1155/2024/6664694)
Supplement: Supplementary 1 — Figure 1: sanger sequencing chromatogram from blood sample. [file 6664694.f1.doc]

**Supplementary Material 1 - Sanger sequencing chromatogram**

**A**


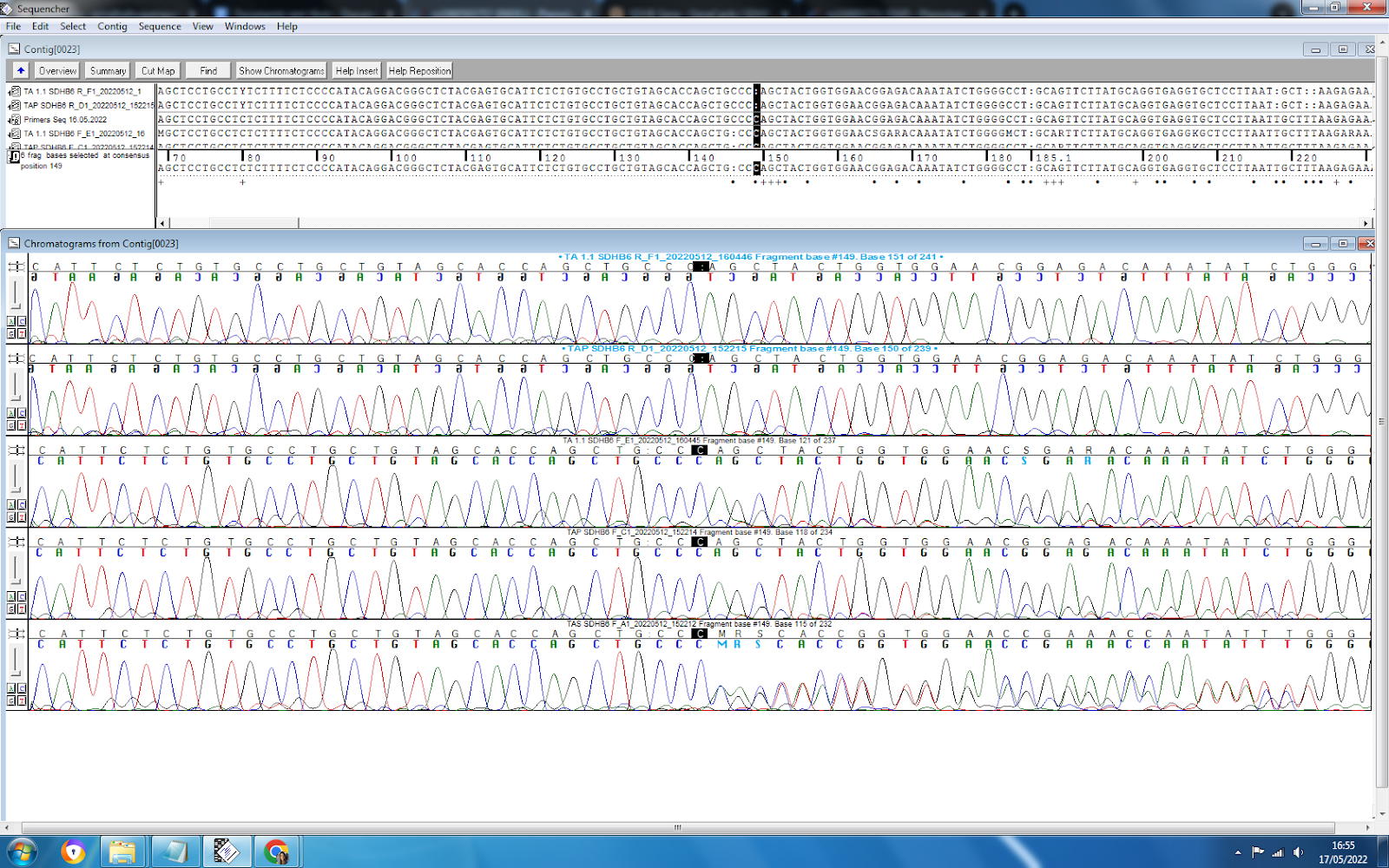


**B**


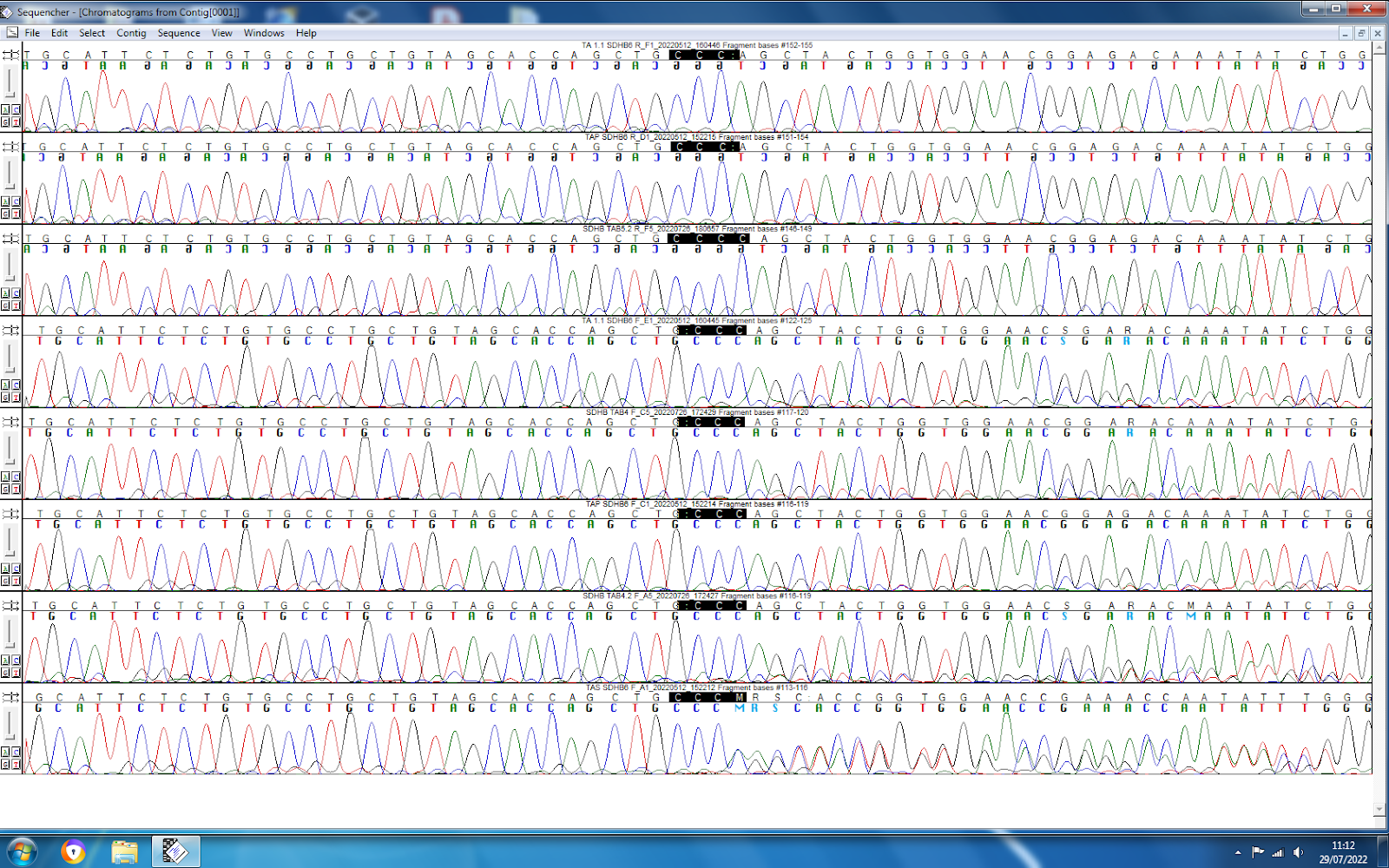


**C**


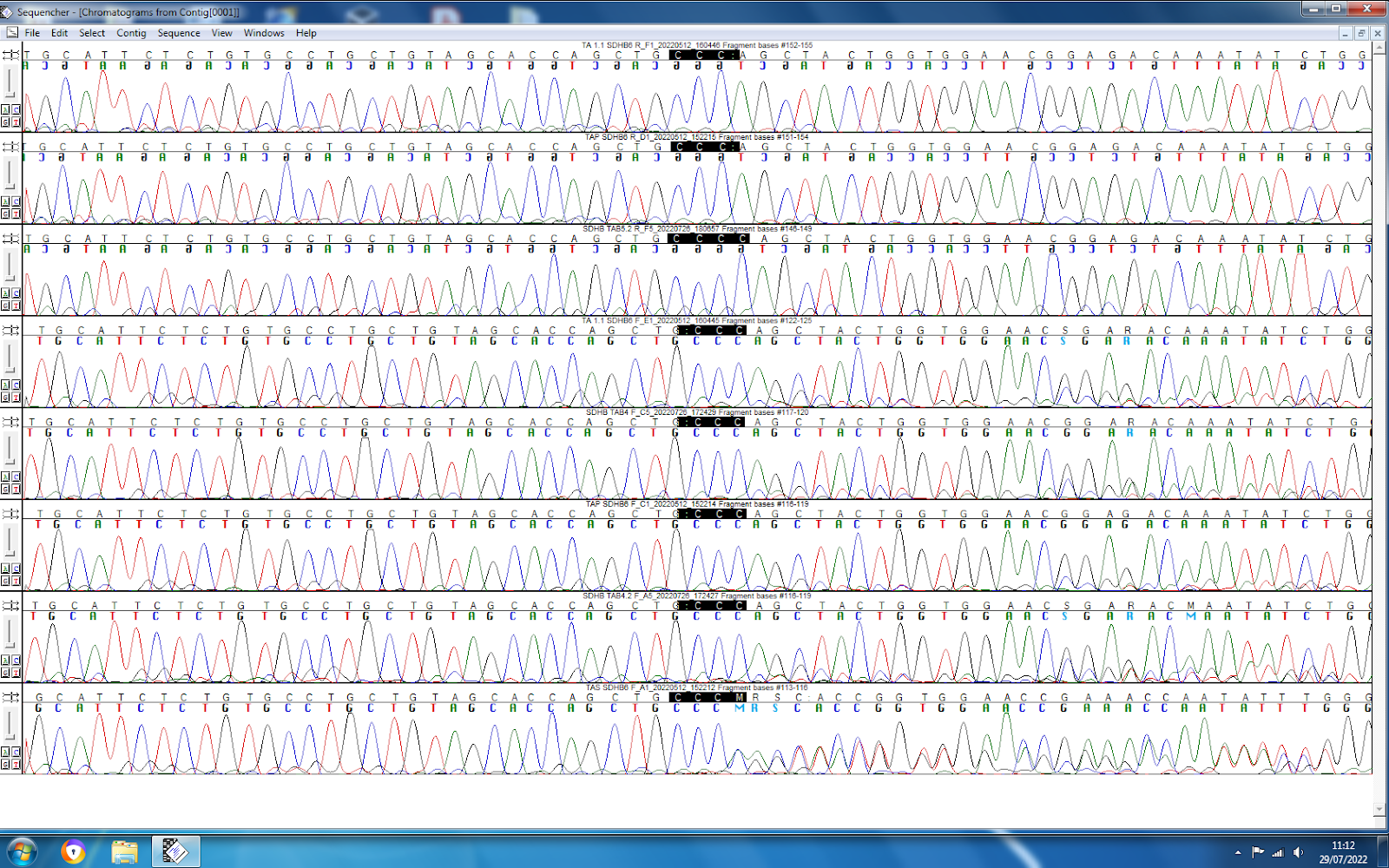


**Supplemental figure 1A:** Sanger sequencing chromatogram from blood sample.

**1B:** Sanger sequencing chromatogram from left adrenal tumor (excised on 2022, DNA extracted from paraffin-embedded tumor tissue).

**1C:** Sanger sequencing chromatogram from psoas Metastasis Tumor (excised on 2022, DNA extracted from fresh tumor tissue).
