# Supplementary material for: From Nonfunctioning Adrenocortical Cancer to Biochemically Silent Paraganglioma Associated with SDHB Mutation: An Uncommon Presentation of a Patient with a Retroperitoneal Mass
Source: Case Rep Endocrinol. 2024 Aug 2;2024:6664694. doi: 10.1155/2024/6664694 (PMC11315972; doi:10.1155/2024/6664694)
Supplement: Supplementary 2 — Figure 2: in silico model of wild-type and mutated SDHB protein. [file 6664694.f2.doc]

**Supplementary Material 2 - In silico model of wild-type SDHB protein**

**
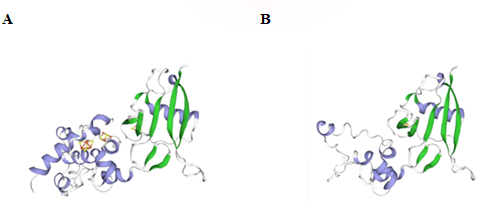
**

**Supplemental figure 2A:** In silico model of wild-type SDHB protein **1B**: In silico model of mutated SDHB protein. SDHB protein sequence was obtained at UniProt [14] and used to build protein model of both the wild-type and predicted pathogenic mutated protein using the Swiss Model Expasy web server ([https://swissmodel.expasy.org/](about:blank), accessed on December 2022)
