# Supplementary material for: From Nonfunctioning Adrenocortical Cancer to Biochemically Silent Paraganglioma Associated with SDHB Mutation: An Uncommon Presentation of a Patient with a Retroperitoneal Mass
Source: Case Rep Endocrinol. 2024 Aug 2;2024:6664694. doi: 10.1155/2024/6664694 (PMC11315972; doi:10.1155/2024/6664694)
Supplement: Supplementary 3 — Figure 3: histological appearance of right retroperitoneal tumor (2014) and right perirenal lesion (2018). [file 6664694.f3.doc]

**Supplementary Material 3: Immunohistochemistry and H&E**

| **Parameter** | **2014** (retroperitoneal tumor) | **2016 (**retroperitoneal tumor **-before slides review)** | **2016 (**retroperitoneal tumor **–after slides review)** | **2018 (**right perirenal lesion**)** | **2018 (**hepatic lesion**)** | **2021** (pulmonary lesion) | **2022** (psoas lesion) |
| --- | --- | --- | --- | --- | --- | --- | --- |
| **Chromogranin-A** | Positive |  | Positive |  |  |  |  |
| **Calretinin** |  |  | Positive | Positive (+) | Positive (+) | Positive (+) | Positive (+) |
| **Cytokeratin** | Positive | Negative |  |  |  |  |  |
| **Enolase** | Positive |  |  |  |  | Positive (++) |  |
| **S100** | Positive |  |  | Positive (+++) |  |  |  |
| **Vimentin** | Positive |  |  |  | Positive (+++) | Positive (+++) |  |
| **Synaptophysin** |  | Negative |  |  |  | Negative |  |
| **Melan A** |  |  | Negative |  | Positive (+) | Positive (+) | Negative |
| **Inhibin-alpha** |  |  | Negative |  | Negative | Negative |  |
| **CD34** |  | Negative |  | Negative |  |  |  |
| **CD117** |  |  |  | Positive (+++) |  |  |  |
| **CD56** |  |  |  |  | Positive (+++) |  |  |
| **PAX 8** |  |  |  |  |  | Negative |  |
| **CD 10** |  |  |  |  |  | Negative |  |
| **TTF.1** |  |  |  |  |  |  | Negative |
| **Ki67** | 3% | 3% |  | 1% |  | 3% | 5% |

**Supplemental Table 1:** Panels of immunohistochemical markers

A

B

**
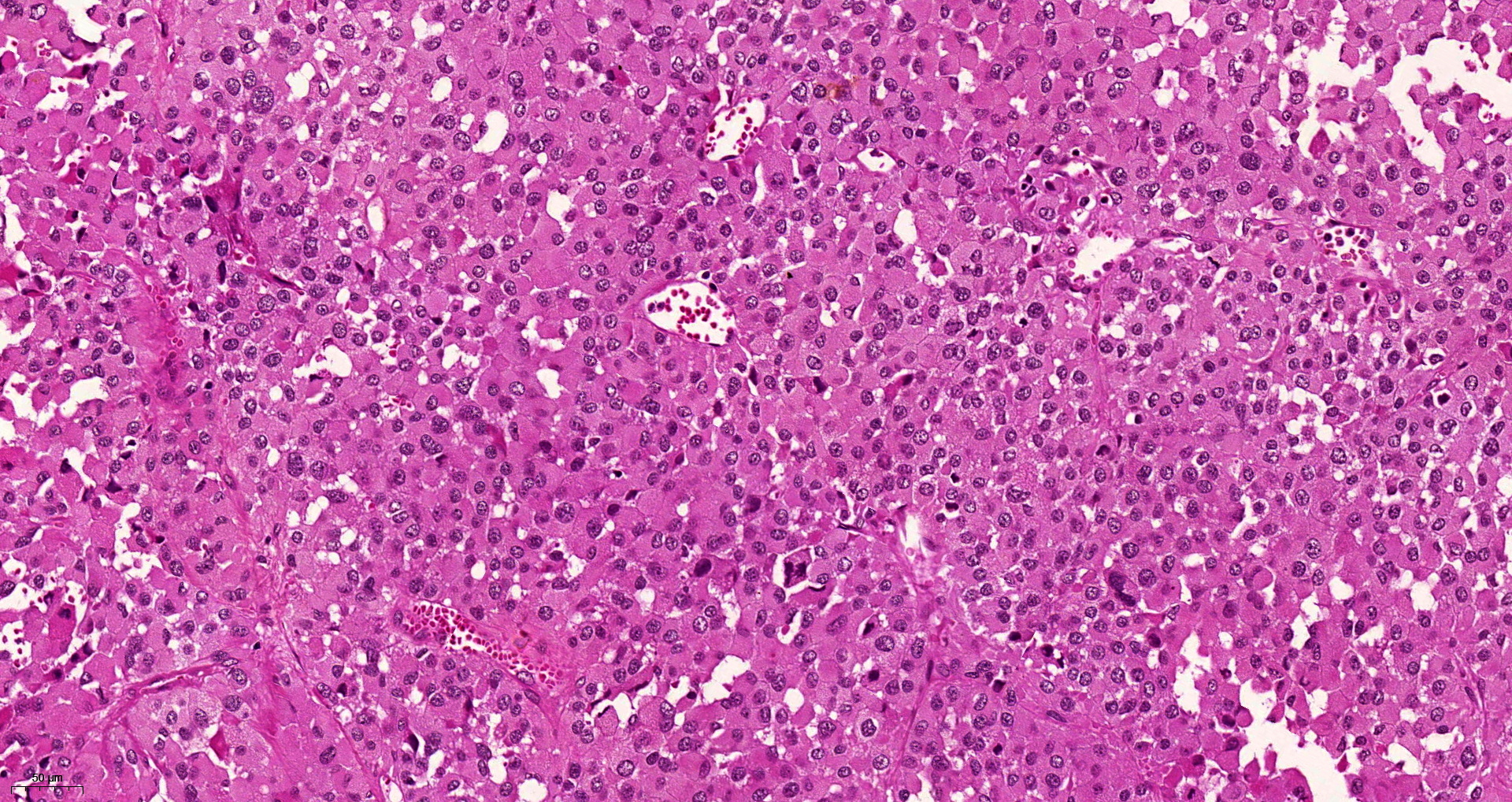
**
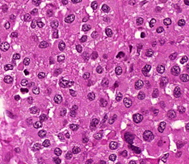


C

D

**
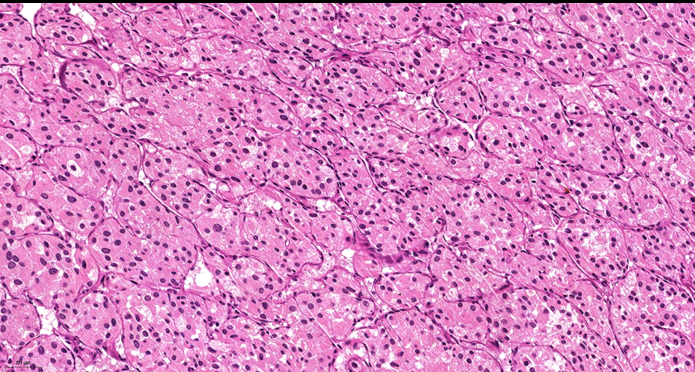
**
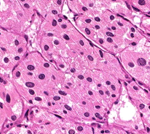


**Supplementary figure 3A:** Histological appearance of right retroperitoneal tumor (2014) with an area of solid appearance, with monotonous proliferation of pleomorphic cells, large eosinophilic cytoplasm and vesicular nuclei. **3B:** Detailed figure 3A.

**3C:** Right perirenal lesion (2018) in a trabecular alveolar arrangement with cells of similar appearance**.** **3D:** Detailed figure 3C.
