# Supplementary material for: From Nonfunctioning Adrenocortical Cancer to Biochemically Silent Paraganglioma Associated with SDHB Mutation: An Uncommon Presentation of a Patient with a Retroperitoneal Mass
Source: Case Rep Endocrinol. 2024 Aug 2;2024:6664694. doi: 10.1155/2024/6664694 (PMC11315972; doi:10.1155/2024/6664694)
Supplement: Supplementary 4 — Table 1: panels of immunohistochemical markers. [file 6664694.f4.docx]

| **Parameter** | **2014** (retroperitoneal tumor) | **2016 (**retroperitoneal tumor **-before slides review)** | **2016 (**retroperitoneal tumor **–after slides review)** | **2018 (**right perirenal lesion**)** | **2018 (**hepatic lesion**)** | **2021** (pulmonary lesion) | **2022** (psoas lesion) |
| --- | --- | --- | --- | --- | --- | --- | --- |
| **Chromogranin-A** | Positive |  | Positive |  |  |  |  |
| **Calretinin** |  |  | Positive | Positive (+) | Positive (+) | Positive (+) | Positive (+) |
| **Cytokeratin** | Positive | Negative |  |  |  |  |  |
| **Enolase** | Positive |  |  |  |  | Positive (++) |  |
| **S100** | Positive |  |  | Positive (+++) |  |  |  |
| **Vimentin** | Positive |  |  |  | Positive (+++) | Positive (+++) |  |
| **Synaptophysin** |  | Negative |  |  |  | Negative |  |
| **Melan A** |  |  | Negative |  | Positive (+) | Positive (+) | Negative |
| **Inhibin-alpha** |  |  | Negative |  | Negative | Negative |  |
| **CD34** |  | Negative |  | Negative |  |  |  |
| **CD117** |  |  |  | Positive (+++) |  |  |  |
| **CD56** |  |  |  |  | Positive (+++) |  |  |
| **PAX 8** |  |  |  |  |  | Negative |  |
| **CD 10** |  |  |  |  |  | Negative |  |
| **TTF.1** |  |  |  |  |  |  | Negative |
| **Ki67** | 3% | 3% |  | 1% |  | 3% | 5% |

**SupplementalTable 1:** Panels of immunohistochemical markers
